# Real-Time Monitoring for Hydraulic States Based on Convolutional Bidirectional LSTM with Attention Mechanism

**DOI:** 10.3390/s20247099

**Published:** 2020-12-11

**Authors:** Kyutae Kim, Jongpil Jeong

**Affiliations:** Department of Smart Factory Convergence, Sungkyunkwan University, 2066 Seobu-ro, Jangan-gu, Suwon 16419, Korea; ryukkt62@skku.edu

**Keywords:** hydraulic system, CNN, bidirectional LSTM, attention mechanism, classification, data augmentation

## Abstract

By monitoring a hydraulic system using artificial intelligence, we can detect anomalous data in a manufacturing workshop. In addition, by analyzing the anomalous data, we can diagnose faults and prevent failures. However, artificial intelligence, especially deep learning, needs to learn much data, and it is often difficult to get enough data at the real manufacturing site. In this paper, we apply augmentation to increase the amount of data. In addition, we propose real-time monitoring based on a deep-learning model that uses convergence of a convolutional neural network (CNN), a bidirectional long short-term memory network (BiLSTM), and an attention mechanism. CNN extracts features from input data, and BiLSTM learns feature information. The learned information is then fed to the sigmoid classifier to find out if it is normal or abnormal. Experimental results show that the proposed model works better than other deep-learning models, such as CNN or long short-term memory (LSTM).

## 1. Introduction

Mechanical fault diagnosis and condition monitoring using artificial intelligence technology are an significant part of the smart factory and fourth industrial revolution [1]. In recent decades, condition monitoring of hydraulic systems has become increasingly important in industry, energy and mobile hydraulic applications as a significant part of the condition-based maintenance strategy, which can reduce machine downtime and maintenance costs and significantly improve planning for the security of the production process. Several sensors are installed to detect errors in the hydraulic system and the data gathered from the sensors can be used to detect faults [2,3].

In recent years, attempts to use deep learning are increasing, especially as the amount of data grow and computing performance improves. Deep learning is applied to a variety of classification problems and has worked well, but requires much data to train. If the amount of data to be trained is insufficient, classification can be inaccurate. However, there is not always enough data in the actual operation of the hydraulic system. Therefore, situations where the amount of data is small should be considered. This problem can be solved by artificially increasing the amount of data. In this paper, we increased data by applying jittering and scaling techniques to existing data [4]. Among deep-learning algorithms, convolutional neural networks (CNNs) have been used for many classification problems and have worked well [5,6]. However, CNN has a problem in extracting too many high-dimensional features. Having many convolutional layers results in a gradient loss, but with too few layers, the network cannot find optimal values [7,8]. Therefore, in this paper we suggest a method having an algorithm that combines CNN and bidirectional long short-term memory networks (BiLSTMs) [9] to monitor the condition of hydraulic systems. The data collected from sensors is first fed into the CNN to extract features and then into the BiLSTM to store long-distance dependency information. Recurrent neural network (RNN) algorithms [10] such as LSTM or Gated Recurrent Units (GRUs) are suitable for sequential data [11,12]. Since the data are sequential, meaningful inferences can be made in both the backward and the forward direction. However, a typical RNN processes only data in the forward direction. Therefore, in this paper, we used BiLSTM, which processes data in both directions. In addition, we added an attention [13] mechanism to properly weight the distribution in relation to the classification results.

Many previous studies have shown that data augmentation techniques can solve the data shortage problem. Perez et al. compared several data augmentation techniques and proposed a new augmentation technique using the Generative Adversarial Network (GAN) [14]. Mikołajczyk et al. proposed a method to augment image data using style transfer and compared it with existing augmentation techniques such as rotation, cropping, and zooming [15]. However, most of the data augmentation techniques are techniques used in the 2D vison domain, and there is very little research on 1D time series data such as hydraulic systems. If data are collected indiscriminately because of lack of data, data with noise or inaccurate labels may be collected, which can degrade the quality of the dataset. This research proposes a method of effectively inflating data by reinterpreting Jittering and Scaling, which are augmentation techniques commonly used in the vision domain, and applying them to 1D time series data. After collecting only accurate and clean data, we can increase the accuracy of determining the condition of the hydraulic system by inflating the amount of data using augmentation techniques. Therefore, this research contributes to solving the data shortage problem by making the small-scale dataset including hydraulic system data useful for real time monitoring in industrial or manufacturing sites.

This paper is organized as follows. Section 2 provides background on neural networks and their learning process. In addition, it introduces the augmentation technique used to increase data. Section 3 introduces the convolutional BiLSTM with the attention model. Section 4 outlines the experiments and discusses the results of the experiments. Section 5 concludes with a summary and presents future research plans.

## 2. Background

This section introduces the basic structure of the neural network and explains how the learning process works. It also introduces the deep-learning architecture used in this paper and reviews how it is used in the condition-monitoring system.

### 2.1. Artificial Neural Networks (ANN)

In 1957, Frank Rosenblatt of Cornell Aeronautical Lab published the perceptron algorithm for learning optimal weights in binary classification problems [16]. Here, binary classification refers to the problem of classifying random sample data into true or false. Neurons have multiple inputs and only one output. Each input is multiplied by its weight. The weights are multiplied by different inputs. The larger this weight value, the more information is conveyed. A constant called bias is added to the sum of the product of the input and the weight. Bias refers to the sensitivity of a neuron in a word. The magnitude of the bias value determines how easily a neuron is excited.

(1) $$u=\sum_{i=1}^{n}\left(w_{i}\times x_{i}\right)+b$$

where *w* is the weight, *x* is the neuron input, and *b* is the bias. If *f* is the activation function and *y* is the output from the neuron, the relationship between the activation function and the output is as follows.

(2) $$\hat{y}=f\left(u\right)=f\left(\sum_{i=1}^{n}\left(w_{i}\times x_{i}\right)+b\right)$$

**Figure 1:**
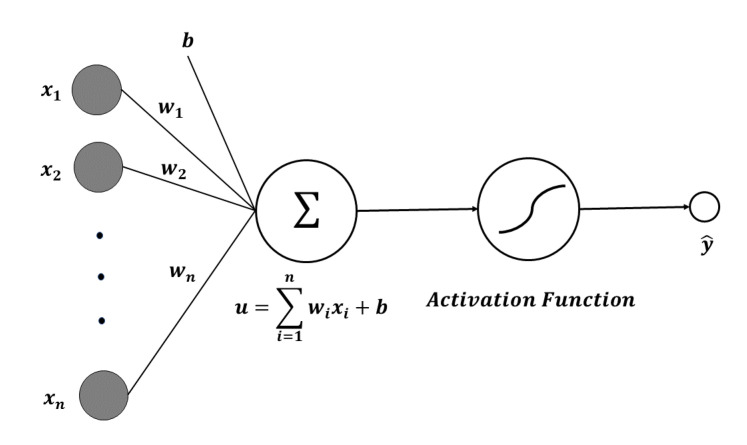
The architecture of perceptron.

As a result of the prediction, $\hat{y}$ is obtained. The error *E* is calculated by the loss function using the actual value *y* and the predicted value $\hat{y}$. The architecture of perceptron is shown in Figure 1.

**Figure 2:**
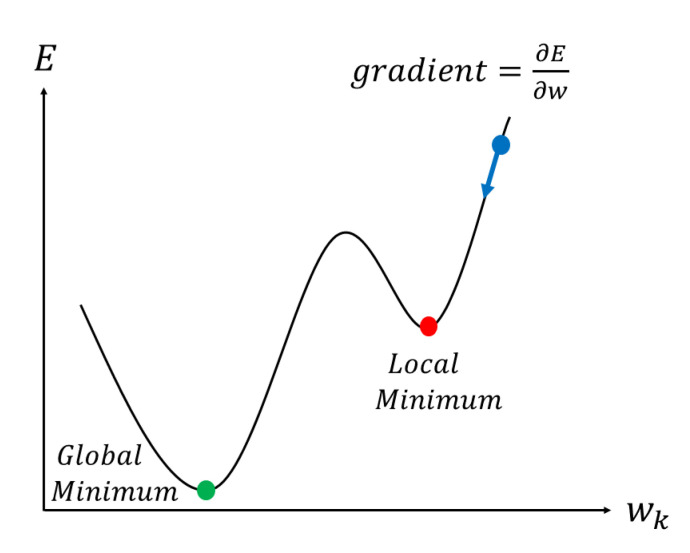
Gradient descent.

In Figure 2, the horizontal axis w is the weight and the vertical axis E is the error. The error depends on the weight, but the weight is gradually changed in the direction where the error is minimized. We can reduce the error little by little by changing all the weights of the neural network so that the error falls [17]. At this time, the amount of change in each weight is determined by the slope of this curve. The same goes for bias [18]. So, to correct all the weights and biases in the neural network, we first need to find the slope of the error for all the weights and biases. Sometimes you fall into a local minimum and can no longer reach the overall minimum without any further weight corrections [19]. This minimum is called the local optimal solution; various adjustments are required to avoid the local optimal solution and obtain the global optimal solution.

**Figure 3:**
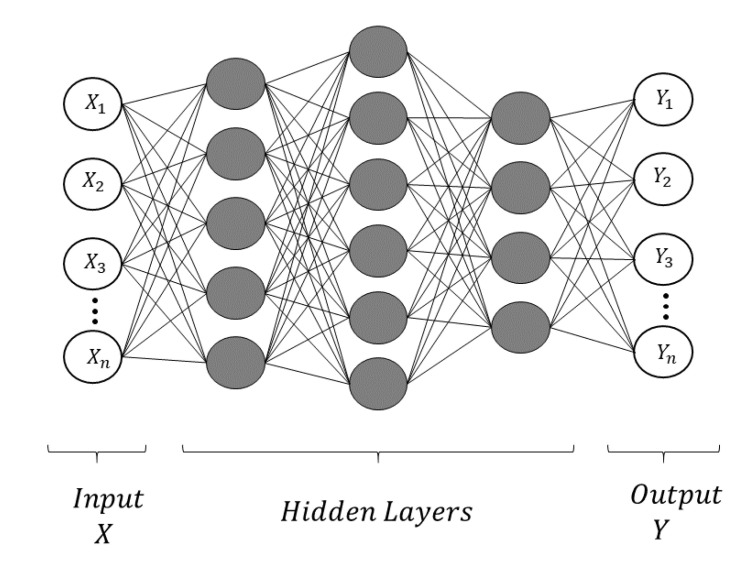
Multi-Layered Perceptron (MLP).

Multi-Layered Perceptron (MLP) is a form of sequentially attaching several layers of perceptrons [20,21]. MLP is also referred to as a feed-forward deep neural network (FFDNN). The layer close to the input is said to be in front, and the layer close to the output is said to be behind. In MLP, there is a connection between perceptrons in adjacent layers, but there is no connection between perceptrons in the same layer [22]. In addition, there is no feedback that leads back to the layer once passed. Layers other than the first input layer and the last output layer are called hidden layers. Figure 3 shows a Multi-Layered Perceptron (MLP) consisting of five layers. The MLP shown in Figure 3 has one input layer, two hidden layers, and one output layer. The unit of the hidden layer is fully connected to the input layer, and the output layer is fully connected to the hidden layer. A network with more than one hidden layer is called a deep artificial neural network.

### 2.2. Convolutional Neural Networks (CNN)

**Figure 4:**
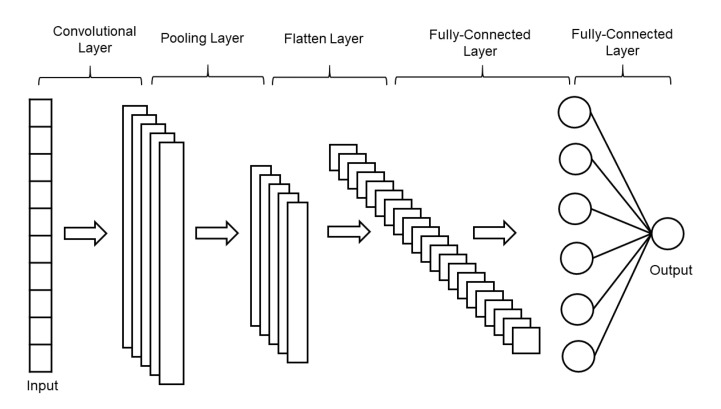
The structure of the one-dimensional convolutional neural network.

The CNN is the most used algorithm for deep learning, a type of machine learning in which models directly classify images, video, text, or sound. CNN learns directly from the data, classifies images using patterns and eliminates the need to manually extract features [23,24]. Depending on the application, you can create a CNN from scratch or use a pre-trained model as a dataset. In general, a CNN consists of several convolutional layers and a pooling layer, also known as a subsampling layer. At the end, one or more fully connected layers follow. A fully connected layer is a multilayer perceptron in which all input units *i* are connected to all output units *j* with weights $w_{ij}$. The subsampling layer, known as the pooling layer, has no parameters to be learned. In other words, there are no weights or intercept units in the pooling layer. Convolutional or fully connected layers have weights and intercepts. The structure of one-dimensional CNN is shown in Figure 4.

### 2.3. Long Short-Term Memory (LSTM)

As the data are transformed through the RNN, some information disappears after every training step. After some time, the state of the RNN has actually no trace of the first input. This can be a serious problem. Several types of cells with long-term memory have been studied to solve this problem. These days, these cells work well, so basic cells are not used a lot. The most popular cells with long-term memory are LSTM [25] cells, which were introduced in 1997 by Hochreiter and Schmidhuber [10] and have gradually been improved over the years thanks to several researchers, including Graves, Sak, and Zaremba [26,27,28]. If you think of an LSTM cell like a black box, it can be used much as a basic cell can be, but it will work much better; that is, training will converge rapidly and detect long-term dependence on the data [29].

Long-term memory $C_{\left(t-1\right)}$ penetrates the network from left to right, passing through the forget gate, losing some memory, and then adding a new piece of memory selected at the input gate with an addition operation. The created $C_{\left(t\right)}$ is sent directly to the output without any further transformation. So at every time step, some memories are deleted and some memories are added. After the addition operation, the long-term state is copied and passed to the tanh function. Then this result is filtered by the output gate to create a short-term state $h_{\left(t\right)}$.

The main layer is the layer that outputs $g_{t}$. This layer is responsible for analyzing the current input $x_{\left(t\right)}$ and the previous short-term state $h_{\left(t-1\right)}$. In LSTM, the output of this layer does not go straight out; instead, the most important part is stored in the long-term state. The three other layers are the gate controller. They use logistic activation functions, so the output ranges from 0 to 1. Their outputs are injected as element-wise multiplication operations, outputting 0 closes the gate and outputting 1 opens the gate. The forget gate decides which of the long-term states should be deleted. The input gate decides which of $g_{t}$ should be added to the long-term state. Last, the output gate decides which of the long-term state should be read and output as h(t) of this time step. In short, LSTM cells learn to recognize critical inputs at the input gate, store them in a long-term state at the forget gate, and extract them whenever necessary. Therefore, LSTM cells work well in catching long-term patterns in time series [30,31].

(3) $$i_{t}=\sigma \left(W_{xi}x_{\left(t\right)}+W_{hi}h_{\left(t-1\right)}+b_{i}\right)$$

here $W_{xi}$, $W_{xf}$, $W_{xo}$, and $W_{xg}$ are the weight matrices of four layers each connected to the input vector **x**.

(4) $$f_{t}=\sigma \left(W_{xf}x_{\left(t\right)}+W_{hf}h_{\left(t-1\right)}+b_{f}\right)$$

here $W_{hi}$, $W_{hf}$, $W_{ho}$, and $W_{hg}$ are the weight matrices of four layers each connected to the previous short-state $h_{\left(t-1\right)}$.

(5) $$o_{t}=\sigma \left(W_{xo}x_{\left(t\right)}+W_{ho}h_{\left(t-1\right)}+b_{o}\right)$$

here $b_{i}$, $b_{f}$, $b_{o}$, $b_{g}$ are biases for each of the four layers.

(6) $$g_{t}=tanh\left(W_{xg}x_{\left(t\right)}+W_{hg}h_{\left(t-1\right)}+b_{g}\right)$$

(7) $$C_{\left(t\right)}=\left(C_{\left(t-1\right)}⊗f_{t}\right)⊕\left(i_{t}⊗g_{t}\right)$$

**Figure 5:**
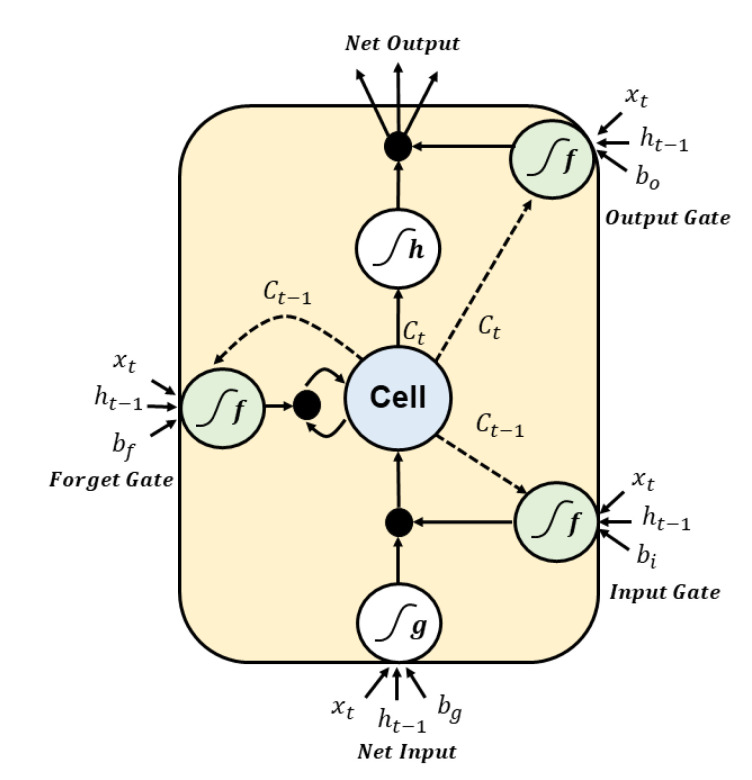
The structure of the long short-term memory (LSTM) neurons.

⊗ means element-wise multiplication and ⊕ means element-wise addition. $b_{i}$, $b_{f}$, $b_{o}$, $b_{g}$ are biases for each of the four layers. The structure of the LSTM neurons is shown in Figure 5.

**Figure 6:**
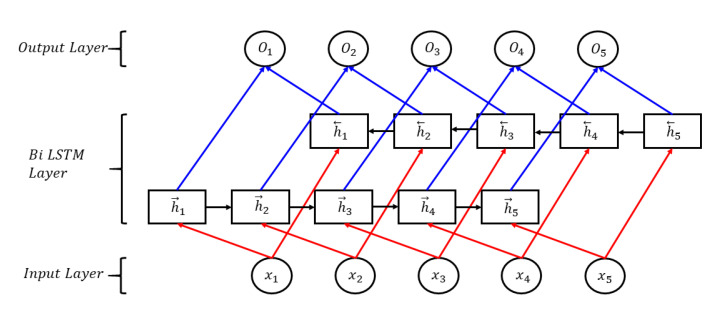
The structure of the bi-directional long short-term memory (BiLSTM) network model.

Invented in 1997 by Schuster and Paliwal, BiLSTM was introduced to make more information available in the network [32]. BiLSTM is connected with two hidden layers in opposite directions. This structure can obtain information from both the previous and the following sequences at the same time [33]. BiLSTM doesn’t need to reconfigure input data and can reach future inputs in the current state. The architecture of the BiLSTM network is shown in Figure 6. The features of the input data are generated by the front of LSTM networks as $\vec{h_{t}}$, and $\overset{\leftarrow }{h_{t}}$ as reverse LSTM networks. Vector $P_{t}$ at time phase t is made by the BiLSTM network. The formula is as follows:

(8) $$\vec{h_{t}}=\vec{LSTM}\left(h_{t-1},x_{t},C_{t-1}\right)$$

(9) $$\overset{\leftarrow }{h_{t}}=\overset{\leftarrow }{LSTM}\left(h_{t+1},x_{t},C_{t+1}\right)$$

(10) $$P_{t}=\left[\vec{h_{t}},\overset{\leftarrow }{h_{t}}\right]$$

### 2.4. Network Model Training

We used a 0.2 dropout to prevent overfitting and used sigmoid as an activation function. The loss function is binary cross entropy, as shown in the equation below.

(11) $$E=-\sum_{n=1}^{N}(t_{n}log\left(y_{n}\right)+\left(1-t_{n}\right)log\left(1-y_{n}\right))$$

The optimization algorithm for weight control is Root Mean Square Propagation (RMSProp). RMSProp is an adaptive learning rate optimizer proposed by Hinton et al. [34]. The size of this slope may differ by weight and may change during learning, making it difficult to select a single global learning rate. RMSProp resolves this by maintaining the moving average of the square gradient and adjusting the weight update to this size. Gradient updates are performed as follows:

(12) $$E{\left[g^{2}\right]}_{t}=\gamma E{\left[g^{2}\right]}_{t-1}+\left(1-\gamma \right){g_{t}}^{2}$$

(13) $$\theta_{t+1}=\theta_{t}-\frac{\eta }{\sqrt{E{\left[g^{2}\right]}_{t}+\epsilon }}g_{t}$$

It is suggested that γ be set to 0.9, whereas a good default value for the learning rate η is 0.001. RMSProp solved Adaptive Gradient Algorithm (AdaGrad)’s shortcomings with a name created by shortening RMSProp. Using exponentially weighted moving averages instead of slope accumulation, long-standing gradients are not reflected in changes in learning rates. RMSProp is an effective and practical optimization algorithm for deep neural network and is one of the main optimization algorithms currently used frequently in deep learning.

### 2.5. Data Augmentation

**Figure 7:**
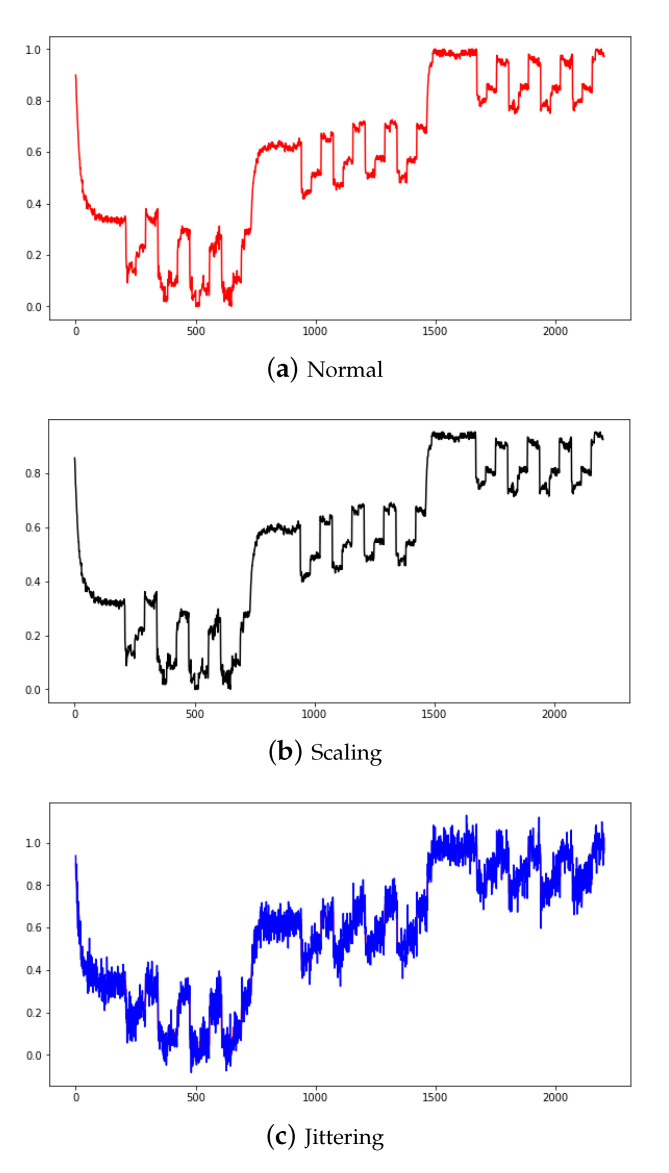
Normal PS1 (**a**), scaling applied PS1 (**b**), jittering applied PS1 (**c**).

**Figure 8:**
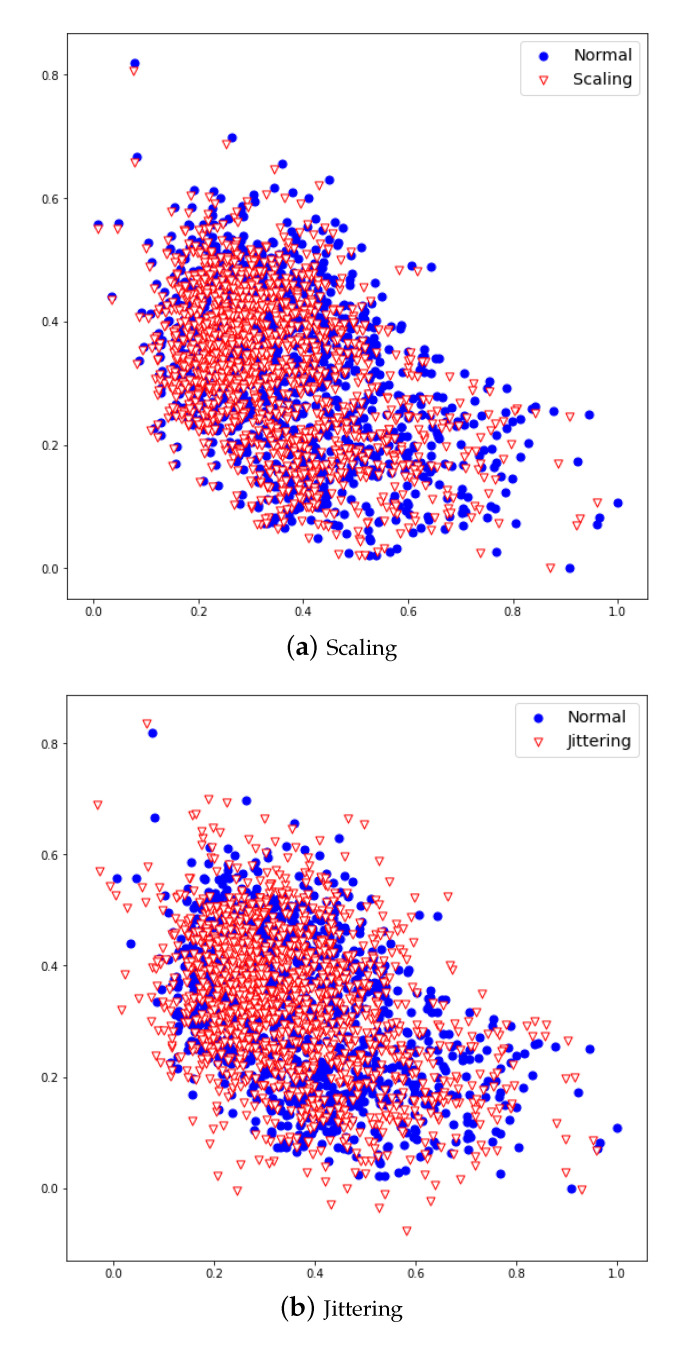
Normal FS1 (**a**), Scaling applied FS1 (**b**), Jittering applied FS1(**c**).

Scaling is a technique that multiplies data by random scalar values, and created new data by resizing it to a range of 10% original data. Jittering is a technique that adds random values [4] and create new data by adding noise to a range 5% of the original data. The data newly created by scaling and jittering are shown in Figure 7a,b. These data augmentation methods can increase robustness and improve performance for multiplication and additive noise. Figure 7 shows the PS1 applied with normal, scaling, and jittering methods. Figure 8 shows the FS1 applied with normal and scaling as a scatter graph.

## 3. Data and Deep-Learning Model

### 3.1. Data Description

In this experiment, we used the binary classification data-set to monitor the state of the hydraulic system. Reference [1] details how to collect data. The data were collected and buffered in the PLC (Beeckhof CS5020) and sent to the PC via EtherCAT. The hydraulic system consists of an operating circuit and a cooling and filtration circuit, each of which is equipped with a range of sensors, including pressure and temperature sensors. Each sensor measures various physical quantities of the circuit, such as pressure (PS1-6), volume flow (FS1-2) temperature (TS1-4), electric pump input power (EPS1), vibration (VS1), and virtual sensors (cooling power, system efficiency, cooling efficiency). A total of 17 sensor signals are collected and are repeated with a predefined working cycle, changing the conditions of the hydraulic components to identify the typical signal pattern of the hydraulic system. Each sensor has a different sample rate range depending on its characteristics and is collected in the range of 100 Hz (e.g., pressure) to 1 Hz (e.g., temperature). The raw sensor features are shown in Table 1.

### 3.2. Deep-Learning Neural Network

We used a model combining CNN and RNN to monitor the state of a hydraulic system and made architecture consisting of two network algorithms of CNN and BiLSTM with an Attention Mechanism. This algorithm effectively resolves the issue of interrelationships between input data in CNN and prevents gradient loss and explosion. The proposed model consists of three parts. First, input data are fed to the model, which uses a one-dimensional CNN primarily for filtering to extract local characteristics from time series data. In the second part, the BiLSTM network stores long term dependent information. Finally, what was learned at the previous layer is fed to the classifier, which uses the sigmoid function to figure out the state of the hydraulic system. Figure 8 show the framework and diagram of the model.

**Figure 9:**
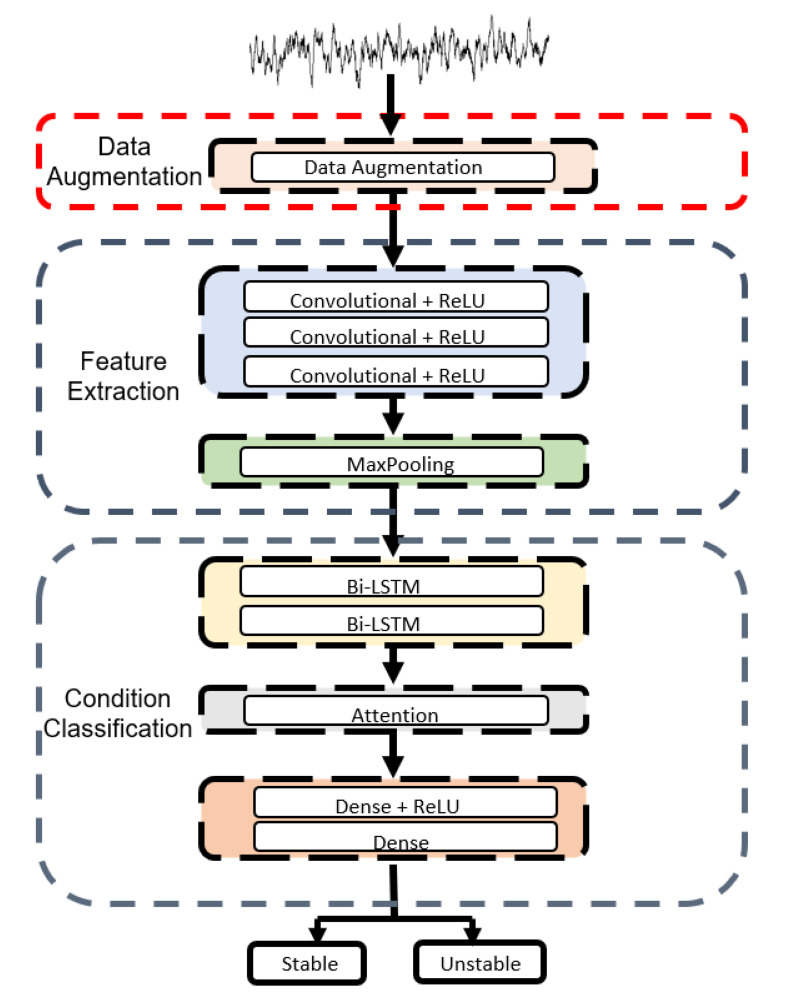
The diagram of the proposed network model.

In the experiment, we used a combined CNN and RNN model to monitor the condition of the hydraulic system. The proposed model consists of three convolutional layers with ReLU as an activation function, followed by maxpooling layer, two BiLSTM layers, attention block and dense layer again. The input of this model is time-series data and constitutes a multi-tensor (2205 × 54) including vibration, temperature, volume flow, and pressure. This model consists of three parts. First, after data is input to the model, local features are extracted from the CNN layers. There are three layers, followed by one maxpooling layer. Maxpooling extracts only the largest value. The second part stores the memory of long distance information in the BiLSTM followed by the attention block. The third part classifies the samples into failure and normal conditions of the hydraulic system using the sigmoid function. Figure 9 shows the diagram of the proposed model.

## 4. Results and Discussion

In this experiment, we used time-series data gathered from sensors installed in the test rig, which consisted of a cooling circuit, filtration circuit, and working circuit and many different values, such as pressure, vibration, and temperature are gathered from the sensors installed. Every sensor had its own sampling rate, which ranged from 1 Hz to 100 Hz [35]. The hardware platform used in this experiment: Intel R CoreTM i7-8700K CPU @ 3.70 GHz and 32 GB of RAM. The software used to test was Tensorflow and Python 3.7.

There were a total of 2205 instances in the hydraulic data set, of which 1449 instances belonged to the negative class (no fault) and 756 instances belonged to the positive class (fault). The validation set consisted of 20% of the training data, and the test data set consisted of 441 samples with 20% of the total data. The proposed model used in the experiment is a model that combines CNN and LSTM, with the attention mechanism added. The model consisted of two one-dimensional convolutional layers, one maxpooling layer, one BiLSTM layer, the attention mechanism, and one fully connected layer of 200 units. At the end, there was a classifier with the sigmoid as an activation function. Detailed parameters are shown in Table 2.

**Figure 10:**
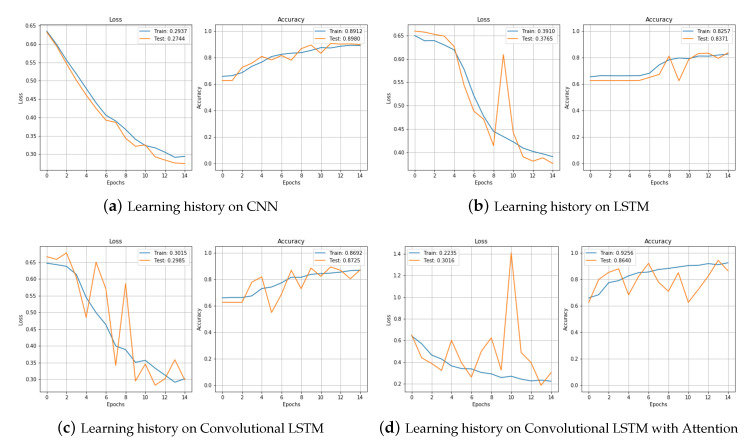
Loss and Accuracy of training set and test set in raw data.

We tested raw data without augmentation, data with jittering, and data with scaling with CNN and LSTM, Convolutional LSTM (CNNLSTM), and the proposed model convolutional bidirectional LSTM with attention (CNNBiLSTM with attention). First, it is the result of fault detection with raw data. Figure 10a shows the process of learning raw data with CNN. Loss gradually decreased and accuracy increased without any problems. We can see that it learned well without overfitting. The final result reached an accuracy of 0.891 in the training set and 0.898 in the test set. Figure 10b is the training result of the model LSTM. In the training set, both loss and accuracy were well trained and the performance was decent, but in the test set, they showed a bouncing appearance. When the epoch was 7, we can see the bouncing in both loss and accuracy, and the final result was also worse than for CNN. In the test set, the loss was 0.376 and the accuracy was 0.837. Figure 10c is the training result of the model CNNLSTM. The training set showed a good learning process, but the test set was very unstable. There were many bouncing spots in the middle, and when the epoch was repeated, the accuracy went up and down. Looking at the process as a graph, it showed the worst shape, but the result in the actual test set was not that bad with a loss of 0.298 and an accuracy of 0.872. Figure 10d shows the learning process of CNNBiLSTM with attention. In the test set, when the epoch was 8 and 10, the loss increased significantly and the accuracy decreased temporarily, but the test results were generally not bad.

**Figure 11:**
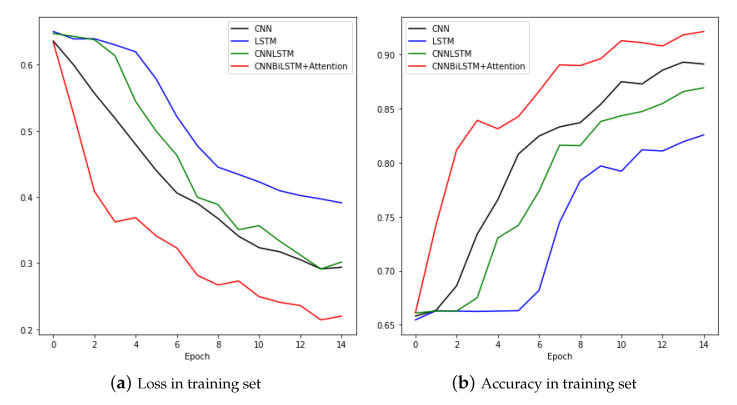
Loss and Accuracy of training set.

Figure 11 compares the training processes of CNN and LSTM, CNNLSTM, and CNNBiLSTM with attention. The CNNBiLSTM with attention model showed the best performance for both loss and accuracy. Results from the test set are shown in Table 3.

**Figure 12:**
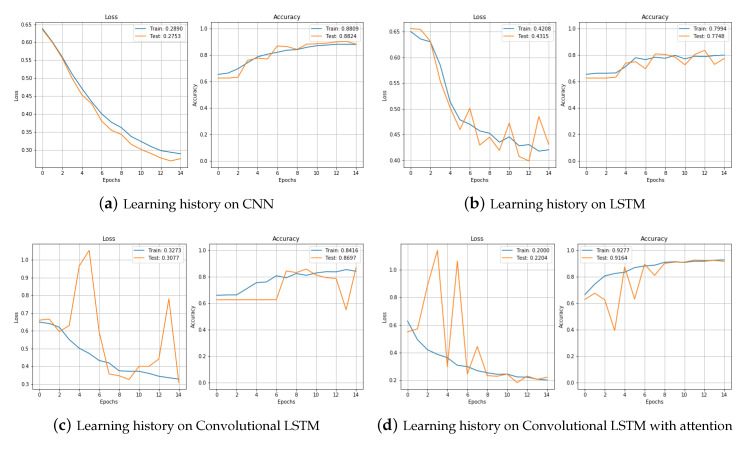
Loss and accuracy of training set in Jittering data.

Figure 12 shows the results of training data augmented by jittering in the training set with CNN, LSTM, CNNLSTM, and CNNBiLSTM with attention and testing in the test set. First, if you look at the training result of CNN, the loss decreased and the accuracy gradually increased as training was repeated. The accuracy was 0.881 in the training set and 0.882 in the test set. The results of learning with the LSTM model are shown in Figure 12b. It looked a little more unstable than when it was trained with CNN. The loss decreased, but the test set was showing a little bouncing. The accuracy was also a little unstable, and was lower than that of CNN. The accuracy on the training set was 0.799 and on the test set was 0.774. The training with the CNNLSTM model produced unstable parts in the validation set, but the accuracy was generally higher than when training with the LSTM model. It was 0.841 in the training set and 0.869 in the test set. Figure 12d is the result of training with the CNNBiLSTM with attention model. In the test set, the loss of the epochs 3, 5, 7 greatly increased, but the accuracy decreased. However, when the training was repeated, it stabilized quickly, and in the end, the accuracy was 0.927 in the training set and 0.916 in the test set.

**Figure 13:**
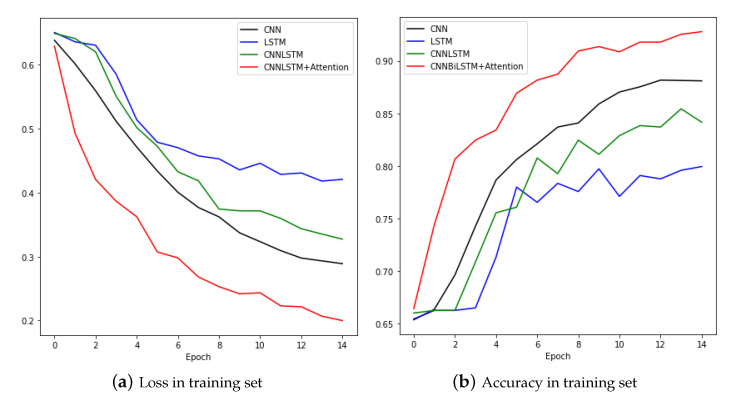
Loss and accuracy of training set and test set in raw data.

Figure 13 compares the training process of the four models. The proposed model, CNNBiLSTM with attention, shows the best performance, and the LSTM model shows the worst performance. Table 4 shows the results from the test set.

**Figure 14:**
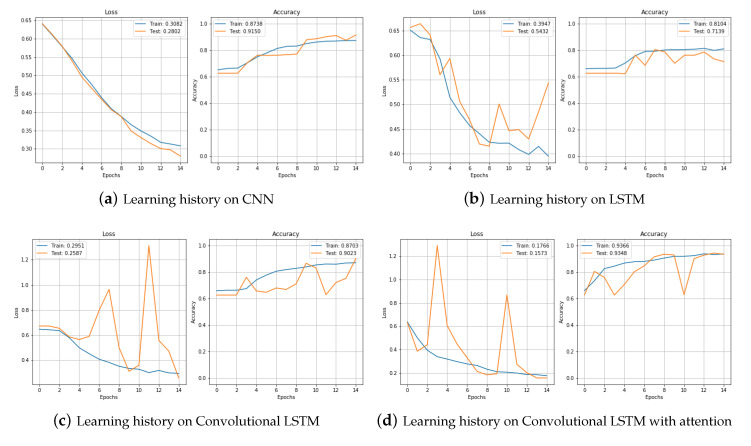
Loss and accuracy of training set and test set in scaling data.

Figure 14 shows the results of training and testing using the CNN, LSTM, CNNLSTM, and CNNBiLSTM with attention after augmentation of data using the scaling technique. First, for the CNN model, the loss fell very quickly and the accuracy increased smoothly. It was 0.873 in the training set and 0.915 in the test set. For the LSTM model, the loss was significantly increased at the end of the test set. For the raw data and data augmented by jittering, LSTM appeared to be unstable. The test showed that the accuracy was 0.713 and the loss was 0.543. For CNNLSTM one can see the loss increase significantly when the epoch was 7 and 11. At this time, you can see that the accuracy also greatly reduced. In the test result, the accuracy was 0.902 and the loss was 0.258. Finally, in the result of training with the CNNBiLSTM with attention model for epochs 3 and 10, the loss increased significantly, but gradually stabilized and finally showed decent results. The final accuracy in the test set was 0.934 and the loss was 0.157.

**Figure 15:**
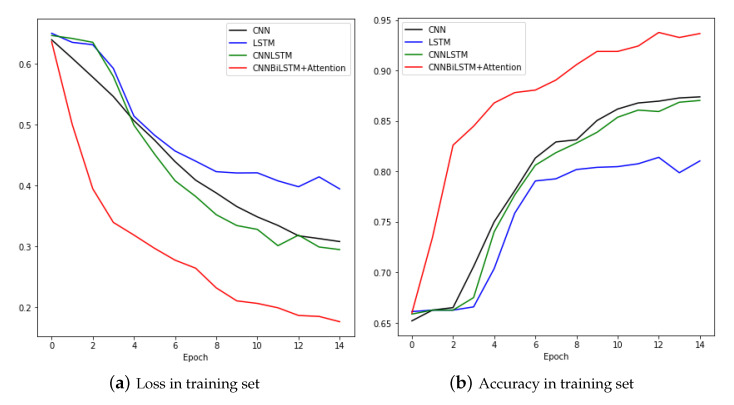
Loss and Accuracy of training set.

Figure 15 and Table 5 compare the training and testing results for the four models. The CNNBiLSTM with attention model showed the best performance, and CNN and CNNLSTM performed similarly. As in the previous case, the LSTM showed the worst performance.

**Figure 16:**
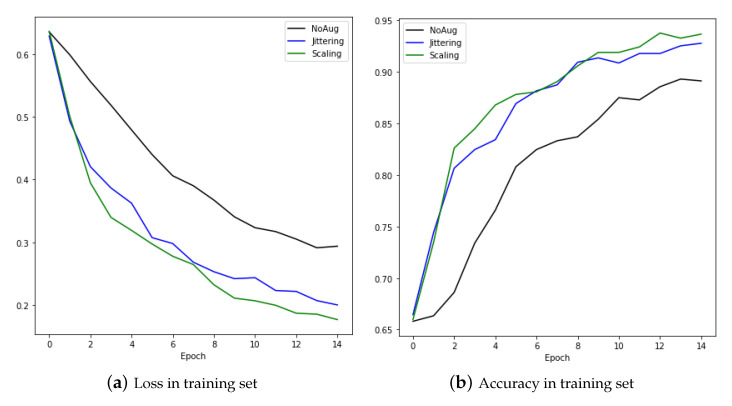
Loss and accuracy in training set.

It can be seen that the performance was better after augmentation of data by means of jittering and scaling than when using raw data. Figure 16 is the result of training with the CNNBiLSTM with attention model. The performance was better when the augmentation technique was applied. The left graph shows the loss as training progresses, and the right graph shows the accuracy.

## 5. Conclusions

Monitoring the condition of the hydraulic system is very important in the industry. When the hydraulic system fails or is unstable, if the condition can be recognized immediately, downtime and repair costs can be reduced, and failure can be prevented in advance. Many sensors were installed on the test bed of the hydraulic system, and data such as temperature and vibration, which are closely related to the failure of the hydraulic system, were collected. However, not always having enough data, and if there are not sufficient data, you may not be able to determine the status properly. So, in this paper, we proposed a data augmentation technique. We created new data using jittering and scaling techniques and increased the amount of data. Through experiments, it has been confirmed that the accuracy is improved by using the data augmentation technique. In addition, it was confirmed that the CNNBiLSTM with Attention Mechanism model performs better than the existing deep learning models such as CNN and LSTM. In future research, we will apply the data augmentation technique not only to hydraulic system data, but also to data from other fields that are widely used in industry. We will also deal with data shortage issues as well as class imbalance issue.

## Figures and Tables

**Table 1:** Description of the hydraulic system dataset sensor features.

Sensor	Physical Quantity	Unit	Sampling Rate
PS1	Pressure	bar	100 Hz
PS2	Pressure	bar	100 Hz
PS3	Pressure	bar	100 Hz
PS4	Pressure	bar	100 Hz
PS5	Pressure	bar	100 Hz
PS6	Pressure	bar	100 Hz
EPS1	Motor power	W	100 Hz
FS1	Volume flow	I/min	10 Hz
FS2	Volume flow	I/min	10 Hz
TS1	Temperature	${}^{\circ }C$	1 Hz
TS2	Temperature	${}^{\circ }C$	1 Hz
TS3	Temperature	${}^{\circ }C$	1 Hz
TS4	Temperature	${}^{\circ }C$	1 Hz
VS1	Vibration	mm/s	1 Hz
CE	Cooling efficiency (virtual)	%	1 Hz
CP	Cooling power (virtual)	kW	1 Hz
SE	Cooling efficiency(virtual)		10 Hz
PS1	Pressure	${}^{\circ }C$	100 Hz

**Table 2:** The parameters of convolutional bidirectional LSTM with attention mechanism.

Layer Name	Output Feature Size	Network
Input Layer	(54, 1)	-
Convolutional layer 1	(52, 64)	Conv1D, kernel size = 3, param = 256
Convolutional layer 2	(50, 64)	Conv1D, kernel size = 3, param = 12,352
Pooling layer	(16, 64)	Maxpooling1D
Bidirectional LSTM layer	(16, 40)	13,600
Dropout	(16, 40)	-
Attention Mechanism	40	-
Fully connected layer 1	200	Dense, param = 8200
Fully connected layer 2	2	Dense, param = 402
Output layer	2	Binary Crossentropy

**Table 3:** Comparison of accuracy and loss.

	CNN	LSTM	CNNLSTM	CNNBiLSTM + Attention
Loss	0.274	0.376	0.298	0.301
Accuracy	0.898	0.837	0.872	0.864

**Table 4:** Loss and accuracy of the test set.

	CNN	LSTM	CNNLSTM	CNNBiLSTM + Attention
Loss	0.275	0.431	0.307	0.220
Accuracy	0.881	0.774	0.869	0.916

**Table 5:** Loss and accuracy of the test set.

	CNN	LSTM	CNNLSTM	CNNBiLSTM + Attention
Loss	0.280	0.543	0.258	0.157
Accuracy	0.915	0.713	0.902	0.934
